# Insights by which TUDCA is a potential therapy against adiposity

**DOI:** 10.3389/fendo.2023.1090039

**Published:** 2023-02-21

**Authors:** Israelle Netto Freitas, Joel Alves da Silva Jr, Kênia Moreno de Oliveira, Bruna Lourençoni Alves, Thiago Dos Reis Araújo, João Paulo Camporez, Everardo Magalhães Carneiro, Ana Paula Davel

**Affiliations:** ^1^ Department of Structural and Functional Biology, Institute of Biology, University of Campinas, Campinas, SP, Brazil; ^2^ Obesity and Comorbidities Research Center, University of Campinas, Campinas, SP, Brazil; ^3^ Department of Physiology, Ribeirao Preto Medical School, University of Sao Paulo, Ribeirao Preto, SP, Brazil

**Keywords:** TUDCA, tauroursodeoxycholic acid, obesity, endoplasmic reticulum (ER) stress, adipose tissue, perivascular adipose tissue, adipocyte

## Abstract

Adipose tissue is an organ with metabolic and endocrine activity. White, brown and ectopic adipose tissues have different structure, location, and function. Adipose tissue regulates energy homeostasis, providing energy in nutrient-deficient conditions and storing it in high-supply conditions. To attend to the high demand for energy storage during obesity, the adipose tissue undergoes morphological, functional and molecular changes. Endoplasmic reticulum (ER) stress has been evidenced as a molecular hallmark of metabolic disorders. In this sense, the ER stress inhibitor tauroursodeoxycholic acid (TUDCA), a bile acid conjugated to taurine with chemical chaperone activity, has emerged as a therapeutic strategy to minimize adipose tissue dysfunction and metabolic alterations associated with obesity. In this review, we highlight the effects of TUDCA and receptors TGR5 and FXR on adipose tissue in the setting of obesity. TUDCA has been demonstrated to limit metabolic disturbs associated to obesity by inhibiting ER stress, inflammation, and apoptosis in adipocytes. The beneficial effect of TUDCA on perivascular adipose tissue (PVAT) function and adiponectin release may be related to cardiovascular protection in obesity, although more studies are needed to clarify the mechanisms. Therefore, TUDCA has emerged as a potential therapeutic strategy for obesity and comorbidities.

## 1 Introduction

Obesity is a global public health problem. Data from 2016 showed that about 1.9 billion adults worldwide were overweight, while 650 million were considered obese (1). Yet, studies indicate that, in 2025, almost 2.3 billion adults will be overweight; and more than 700 million will be obese (2). About public expenses with health, it is postulated that the costs of caring for obese patients will double by 2050 (US$10.1 billion) compared to 2010 (US$5.8 billion) (3). According to the Global Burden of Disease study, 4.7 million people died prematurely in 2017 as a result of obesity (4). Obesity is a risk factor for developing many disorders such as diabetes mellitus, hypertension, cardiovascular events, obstructive sleep apnea syndrome, cancer, and musculoskeletal diseases (5). Obesity also has a negative impact on quality of life and increases the costs of healthcare (6).

Adipose tissue is an endocrine organ with high metabolic activity. It corresponds to 20-28% of the body mass of healthy individuals and may represent up to 80% of the body mass in obese individuals (7). It is a specialized connective tissue composed predominantly by adipocytes and separated by a thin layer of extracellular matrix (8). It was believed that adipose tissue operated only as an energy store in the form of triglycerides, however, this tissue also works as an endocrine organ capable of secreting numerous hormones and adipokines that contribute to energy homeostasis (9). Besides these metabolism-related functions, adipose tissue regulates other physiological processes related to reproduction, immunity, angiogenesis, extracellular matrix restructuring, steroid metabolism, and body temperature (7).

White adipose tissue (WAT) is composed primarily of white and a few beige adipocytes, depending on its location. White adipocytes have a vacuole with a single lipid droplet and few cellular organelles and can vary in size according to the amount of stored triglycerides. Its main function is energy storage, but WAT also secretes a wide variety of adipokines, such as leptin and adiponectin, which are the first two proteins discovered (10). In addition, beige or brite adipocytes are generally found scattered amongst the white adipocytes and have the potential to generate heat when facing cold exposure or adrenergic receptors stimulation (11, 12). On the other hand, brown adipose tissue (BAT) adipocytes present several lipid droplets vacuolized and a large number of mitochondria, which gives the tissue a brown color. Its main function is the production of energy in the form of heat, a process accomplished by the uncoupling protein-1 (UCP-1), which is considered its phenotypic characteristic protein (10, 13).

Adipose tissues regulate energy homeostasis, providing energy in nutrient-deficient conditions and storing it in high-supply conditions (14). However, when the excess of stored energy exceeds the expenditure, obesity can be established (15). Therefore, studies involving the comprehension, prevention, and treatment of obesity are extremely important. Here, we review endoplasmic reticulum (ER) stress as a mechanism involved in adipose tissue morphological, functional and molecular changes on the setting of obesity highlighting recent advances in therapeutic actions of the tauroursodeoxycholic bile acid (TUDCA).

## 2 Changes in adipose tissue depots in obesity: A role for ER stress

### 2.1 ER stress

The ER is an important organelle of eukaryotic cells responsible for maintaining calcium homeostasis, for the biosynthesis of phospholipids and for the synthesis and folding of proteins that will be directed to the plasma membrane or to secretory vesicles (16). However, inflammation, nutrient deprivation, hypoxia, acidosis, and oxidative stress can disturb the homeostasis of ER leading to ER stress (17–19). ER stress is established when an imbalance occurs between the demand and the capacity of the ER for protein folding, resulting in the accumulation of misfolded/unfolded proteins. To normalize ER homeostasis and reestablish protein folding, cells rely on a defense mechanism called unfolded protein response (UPR) (20, 21).

UPR monitors protein folding in the ER and adjusts folding capacity to match the amount of synthesis. For this, three sensors that are present in the ER membrane are required: protein kinase R-like ER kinase (PERK), transcription factor 6 (ATF6), and inositol requiring enzyme 1α (IRE1α) (20, 21). Under physiological conditions, these sensors are bonded to a chaperone protein named as glucose-regulated protein 78 (GRP78) an ER chaperone also referred to as the immunoglobulin heavy chain binding protein (BiP), which keeps them inactivated. However, when levels of misfolded proteins increase, BiP dissociates from PERK, ATF6 and IRE1α activating three distinct signaling pathways (22). When BiP dissociates from IRE1α, IRE1α is autophosphorylated and activates the transcription factor X Box Protein 1 (XBP-1) that regulates the expression of chaperones and enzymes involved in the degradation of misfolded proteins arising from the ER (23, 24). PERK phosphorylates eukaryotic initiation factor 2 (eIF2α) and induces ATF4 translation, one of the UPR-dependent signaling proteins. In general, these pathways regulate protein synthesis rate, biosynthesis of new chaperones, protein trafficking within the ER, protein degradation, and finally, apoptosis, if ER homeostasis is not reestablished (20–22).

ER stress is linked to oxidative stress (23) as protein misfolding produces reactive oxygen species (ROS) (24, 25) that further impairs protein folding and depletes ER Ca^2+^ levels, aggravating ER stress (26, 27). In addition, IRE1α/XBP-1 and PERK/eIF2α induce the expression of C/EBP homologous protein (CHOP) that contribute to ROS generation and apoptosis (28).

### 2.2 ER stress in WAT and BAT during obesity

ER stress has been associated with dysfunctional WAT and BAT during obesity. In WAT, high-fat diet (HFD)-induced obesity enhances expression of UPR markers such as PERK, ATF-6, IRE1-α, ATF-4, and CHOP (29–31). Furthermore, ER stress affects adipokine secretion and action by impairing adiponectin synthesis and secretion (30, 31) and by inducing leptin resistance (32). Lipogenesis and adipogenesis are also modulated by ER stress (33). In line with this, activation of ER stress increases triglycerides, SREBP-1c, and FAS in human mature adipocytes (34). CHOP upregulation limits beige adipocyte differentiation by inhibiting UCP-1, Cox8b, Cidea, Prdm16, and PGC-1α (35), while CHOP deficiency upregulates PPARγ and adiponectin (29). IRE1α impedes beige fat activation in white and beige adipocytes, degrades PGC1-α mRNA, and limits thermogenesis (36).

During obesity, ER stress can be a mechanism potentiating proinflammatory state in WAT and UPR pathways converge with inflammatory signaling pathways (37, 38). CHOP deficiency enhances M2 macrophages abrogating WAT inflammation (29). In addition, IRE1α favors M1/M2 adipose tissue macrophages polarization and impairs WAT browning (30). Therefore, ER stress can modulate WAT adipocytes morphology and WAT inflammation in the setting of obesity.

In humans and in animal models of obesity, density and thermogenic activity of brown adipocytes is lower, as they go through a process called whitening, due but not limited to the excess of lipids (39, 40). Enlarged white-like brown adipocytes may present accumulation of large lipid droplets, mitochondrial dysfunction, and oxidative stress (41–44). The inflammatory profile triggered in BAT during obesity reduces the expression of UCP-1 and BAT thermogenic activity. Furthermore, inflammatory mediators have been shown to prevent the expansion of BAT in obesity, by promoting cell apoptosis *via* tumor necrosis factor-α (TNF-α) or reducing tissue proliferation, inhibiting catecholamine signaling (45).

In BAT of obese mice, genes related to ER stress and UPR such as GRP78, CHOP, ATF4 and ATF6 are enhanced while thermogenic genes UCP-1 and PGC1-α are reduced; induction of ER stress resulted in brown adipocytes apoptosis *in vitro* and *in vivo* (46). UCP-1 deficiency increases CHOP and XBP1 transcript levels and eIF2α phosphorylation, linking deficient thermogenesis to ER stress in BAT (47). However, the role of UPR response elements in BAT are still under investigation. The IRE1α/XBP1 pathway seem to be highly activated compared with other UPR branches during the induction of UCP-1 transcription in BAT, and its induction mechanism is independent of ER stress (48). Also, in brown adipocytes, PERK has a function independent of UPR, and seems to be essential for mitochondrial thermogenesis (49). Therefore, the role of ER stress in BAT in response to metabolic challenges as obesity still needs to be clarified.

### 2.3 Ectopic fat

In addition to classic fat depots, adipose tissue can accumulate ectopically during the development of obesity, which can contribute to impaired function in the deposited tissues (50), such as blood vessels and liver (51). In this sense, it is known that a progressive increase in the deposition of lipids in the liver leads to inflammation, hepatocellular degeneration, and collagen deposition, resulting in non-alcoholic steatohepatitis (NASH) (52), which, if not reversed, leads to irreversible liver cirrhosis and also increases the risk for the occurrence of hepatocellular carcinoma (53, 54). ER stress and UPR pathways are molecular mechanisms associated with NASH. CHOP, Bip, IRE1α and XBP1 are involved in hepatic lipid metabolism (55). In the livers of obese mice, ER stress associated with IRE1α and PERK activation, and CHOP upregulation activate the NLRP3 inflammasome and induce hepatocyte inflammation and apoptosis (56). Therefore, long-term ER stress leads can result in liver injury in obesity. The importance of ER stress to hepatic lipid metabolism in obesity and NASH was the topic of a recent review (57).

The obesity has also been demonstrated to alter the perivascular adipose tissue (PVAT) (58, 59). PVAT presents morphological characteristics that vary according to the vascular bed, resembling WAT in mesenteric arteries and abdominal aorta or BAT/beige in thoracic aorta (60–62). PVAT synthesizes and releases a variety of vasoactive substances that paracrinally influence peripheral vascular resistance and, therefore, blood pressure. PVAT may lose its vasoregulatory capacity due to a decrease in the release of vasodilating adipokines (63, 64). The expansion of PVAT in obesity is associated with immune cells infiltration, reduced adiponectin and increased leptin secretion, greater ROS production, and inducible nitric oxide synthase (iNOS) expression (63–67). In PVAT depots, ER stress is associated with the expression of pro-inflammatory factors including NF-kB, impaired vascular function (vasodilation and contraction) and atherosclerotic plaque destabilization (68, 69).

Given the metabolic and cardiovascular importance of PVAT and other ectopic fat depots, further studies need to be conducted in order to better elucidate the impact and pathophysiological of obesogenic diets and ER stress in ectopic fat. It is also relevant to identify pharmacological tools targeting ER stress in PVAT that could be a coadjuvant approach to the treatment and prevention of vascular complications associated with cardiometabolic diseases.

## 3 Actions of TUDCA in adiposity

### 3.1 Molecular mechanisms of TUDCA

Bile acids (BAs) are synthesized in hepatocytes by the enzyme cholesterol 7α-hydroxylase (CYP7A1) from cholesterol, generating primary BAs such as cholic acid (CA) and chenodeoxycholic acid (CDCA). In the intestine, through deconjugation, oxidation and epimerization reactions carried out by the intestinal microbiota, primary BAs give rise to secondary BAs such as deoxycholic acid (DCA), lithocholic acid (LCA) and ursodeoxycholic acid (UDCA). After the formation of secondary BAs, some BAs are conjugated in hepatocytes with the amino acids glycine as glycocholic acid (GCA), or taurine, as TUDCA, which makes these compounds obtain greater solubility and ionization in the intestinal lumen (70, 71).

At first, it was believed that BAs served only to assist in the process of digestion of lipids and fat-soluble vitamins (71, 72). However, recent data show that these molecules can regulate lipid and glycemic metabolism, BA synthesis, and the immune system, presenting metabolic and endocrine functions (71–73). These effects result from signaling through receptors such as the Farnesoid X receptor (FXR), the G protein-coupled BA receptor (TGR5) and Sphingosine-1-phosphate receptor 2 (S1PR2) (72–74). FXR is a nuclear receptor while TGR5 and S1PR2 are membrane receptors. These three receptors are classically expressed in the liver and digestive tract, but also have been identified in other tissues such as the heart, blood vessels, and adipocytes (75, 76). Additionally, preadipocytes and mature adipocytes express other factors involved in BA metabolism, such as BSEP (bile salt exporting pump), a hepatic protein that functions as a bile salt export pump in liver cells mediating BA transport into the bile canaliculi (77, 78). BAs conjugation with taurine increases affinity to the membrane and nuclear receptors (75).

Inhibition of ER stress could be a therapeutic intervention against morphofunctional alterations of overall adipose tissue depots in obesity. In this sense, the ER stress inhibitor TUDCA, a BA conjugated to taurine with chemical chaperone activity, has emerged as a therapeutic strategy to minimize adipose tissue dysfunction and metabolic alterations associated with obesity (79, 80).

### 3.2 Effects of TUDCA and its receptors in obesity

TUDCA was recently demonstrated to be an important mediator of beneficial metabolic effects on diet-induced obesity models, as by activating FXR and TGR5, taurine-conjugated BAs can improve glucose homeostasis, lipid metabolism and BAT thermogenesis (81, 82).

The activation of TGR5 increases energy expenditure in HFD-induced obese mice, limiting obesity and insulin resistance (83). TGR5 signals through cyclic adenosine monophosphate/protein kinase A (cAMP/PKA) and deiodinase-2 activation in mouse and human brown adipocytes and increased thermogenic activity (84–86). This pathway can also mediate beiging of WAT by increasing mitochondrial content and fission in white adipocytes from HFD mice (85). In BAT, TGR-5 induces thermogenesis (83). FXR activation can reduce both weight gain and inflammatory markers in WAT of HFD-induced obese mice, as well as limit the expansion of WAT under obesogenic conditions (87–89). Therefore, BA receptors activation seems to limit lipid accumulation, inflammation and metabolic changes in WAT during obesity. However, it was demonstrated that FXR-deficiency can attenuate WAT expansion, body weight and insulin resistance in mouse models of genetic and diet-induced obesity (90). Controversial data is also observed in ectopic fat as reduced or increased fat liver accumulation was observed following FXR activation (88, 89). Therefore, further studies are needed to clear the molecular mechanism of BA receptors in adipocytes during obesity.

Our group has demonstrated that TUDCA has a beneficial effect on glucose-induced insulin secretion through TGR5/PKA signaling in beta cells (91). Interestingly, increased insulin secretion is induced by TUDCA in isolated pancreatic islets under thapsigargin-induced ER stress (72). Therefore, it is likely that ER stress inhibition in beta cells is involved in TUDCA effects. In agreement, by modulating ER stress, administration of TUDCA in obese and type 2 diabetic mice normalized glycemia, restored insulin sensitivity in liver, muscle and adipose tissues, as well as reduced fatty liver disease (92). Of note, TUDCA may act as an insulin receptor (IR) agonist, which, in addition, can contribute to its beneficial effects on insulin sensitivity (93).

**Figure 1:**
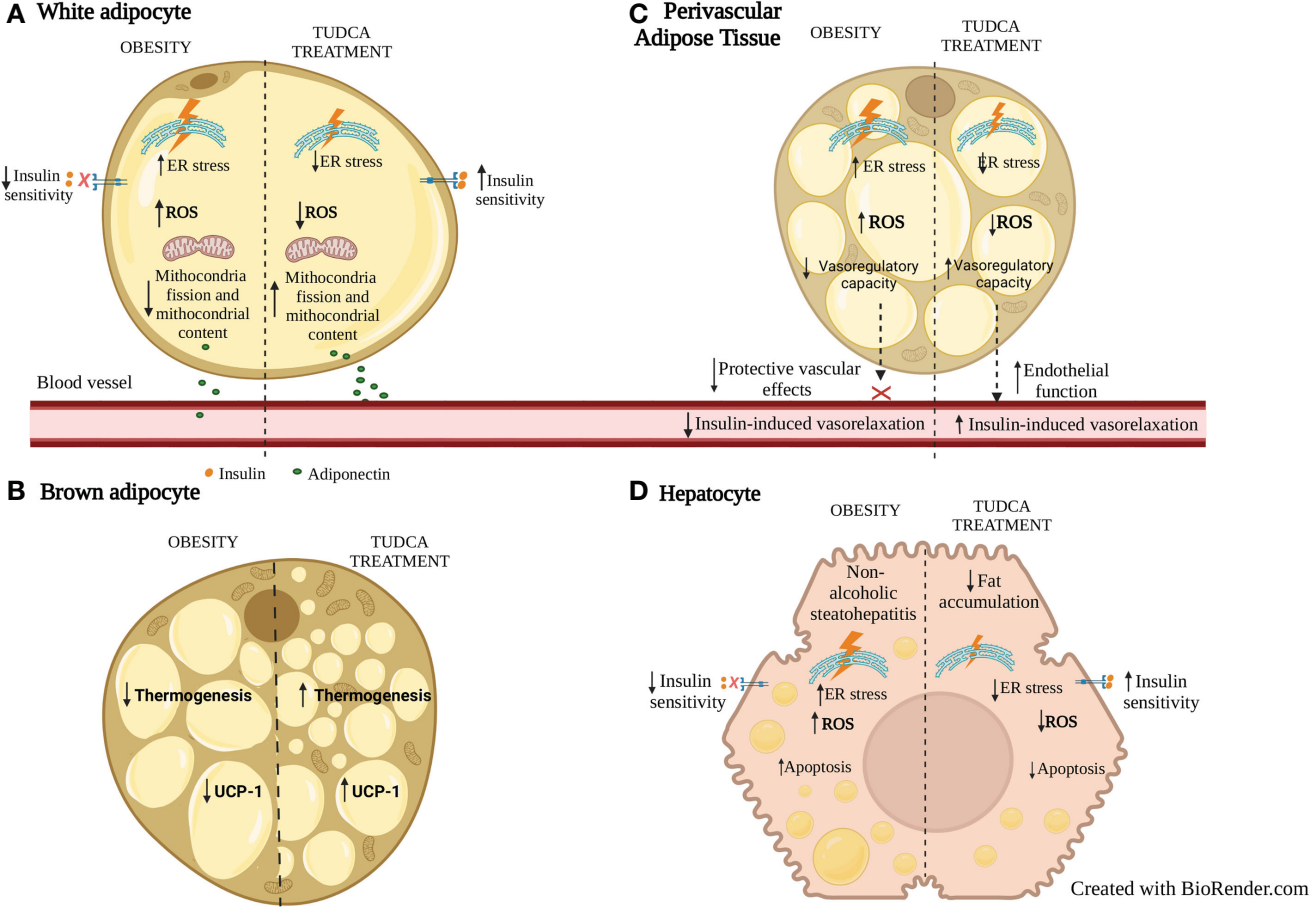
Effects of TUDCA treatment on WAT, BAT, PVAT and liver fat during obesity. **(A)** In WAT adipocytes, ER stress inhibition with TUDCA is associated with increased mitochondrial fission and content, decreased ROS, and increased insulin sensitivity and adiponectin secretion. **(B)** In BAT, TUDCA increases thermogenesis and expression of UCP-1. **(C)** In PVAT, ER stress inhibition with TUDCA is associated with decreased ROS, increased anticontractile and endothelial function. **(D)** In hepatocytes, in addition to inhibiting ER and oxidative stress, TUDCA treatment increases insulin sensitivity, decreases fat accumulation and adipocyte apoptosis. ER: endoplasmic reticulum; ROS: reactive oxygen species. UCP-1: uncoupling protein-1.

TUDCA treatment of human adipose derived stem cells (hASCs) significantly decreases ER stress marker GRP78, adipogenic markers such as PPARγ and glycerol-3-phosphate dehydrogenase 1 (GPDH), and lipid accumulation (94) suggesting that ER inhibition by TUDCA is associated with decreased adipogenesis. Such actions of TUDCA were similar to the ER stress inhibitor 4-phenyl butyric acid (PBA). Further, TUDCA or PBA treatment decreased ER stress markers, free cholesterol, inflammatory cytokines level, and NF-kB activity in WAT of HFD-induced obese mice (37). Finally, ER stress inhibition induced by TUDCA is associated with the inhibition of ROS production and attenuated cleaved-caspase-3 expression, resulting in an antiapoptotic effect in white adipocytes exposed to high glucose (95). In BAT, ER stress inhibition is associated with increased UCP-1 and thermogenesis (96, 97). The major effects of TUDCA on WAT and BAT are summarized in **Figure 1**.

TUDCA treatment also has been suggested as beneficial for ectopic fat during obesity (**Figure 1**). Reduced ER stress markers PERK and IRE-1α phosphorylation is demonstrated in the liver of diabetic ob/ob mice associated with resolution of fat liver disease (92). TUDCA can limit hepatocyte lipoapoptosis by suppressing phosphorylation of eIF2α, XBP1 splicing, BiP and ATF4 expression (98). TUDCA may also have beneficial effects on PVAT. In line with this, TUDCA reduced ER stress markers GRP78 and ATF4 on PVAT from type 2 diabetic mice which was associated with improved endothelial function and reduced vascular stiffness (99). The ex vivo exposure to palmitate or thapsigargin both impaired insulin-induced vasorelaxation in the aorta which was prevented by TUDCA, associated with reduced expression of ER markers IRE1α and eIF-2α phosphorylation in PVAT (100). However, the mechanisms associated with TUDCA-induced PVAT mediated protective vascular effects still need to be addressed. As TUDCA increases adiponectin production in obese mice and in adipocytes (76, 101) this can be an additional beneficial mechanism associated with cardiovascular protection. Future studies need to be addressed to elucidate mechanisms of metabolic and vascular effects of TUDCA, focused on the involvement of ER stress inhibition and/or direct effects of this BA.

## 4 Conclusion

Here, we summarize the main findings on the effects of TUDCA and receptors TGR5 and FXR on adipose tissue in the setting of obesity. TUDCA has been demonstrated to limit metabolic disturbs associated with obesity by inhibiting lipid accumulation, ER stress, inflammation, and apoptosis in adipocytes. TUDCA also improves insulin sensitivity in obese mice and may act as an IR agonist. The beneficial effect of TUDCA on PVAT function and adiponectin release may be related to cardiovascular protection in obesity, although more studies are needed to clarify the mechanisms. A limitation of the studies is to identify possible direct effects of TUDCA independent of ER stress inhibition, which are interesting for elucidating possible therapeutic targets in obesity and other diseases.

As summarized in this review, TUDCA can be considered a multi-targeted therapy, as this BA modulates glucose and lipid metabolism, inflammatory response, adipogenesis, macrophage differentiation and other general metabolic responses. TUDCA has been approved by the US administration for clinical use in diseases such as cholelithiasis and cholestatic liver disease (102, 103). Therefore, it may be suggested that TUDCA treatment could be also an important therapeutic agent for obesity and comorbidities.

## Author contributions

IF and AD contributed to the study conception, design and writing. JS, BA, TR, JC, and EC contributed to the writing and reviewing. KO made the figures. All authors contributed to the article and approved the submitted version.
